# Algorithmic Versus Expert Human Interpretation of Instantaneous Wave-Free Ratio Coronary Pressure-Wire Pull Back Data

**DOI:** 10.1016/j.jcin.2019.05.025

**Published:** 2019-07-22

**Authors:** Christopher M. Cook, Takayuki Warisawa, James P. Howard, Thomas R. Keeble, Juan F. Iglesias, Erick Schampaert, Ravinay Bhindi, Alphonse Ambrosia, Hitoshi Matsuo, Hidetaka Nishina, Yuetsu Kikuta, Yasutsugu Shiono, Masafumi Nakayama, Shunichi Doi, Manabu Takai, Sonoka Goto, Yohei Yakuta, Kenichi Karube, Yoshihiro J. Akashi, Gerald J. Clesham, Paul A. Kelly, John R. Davies, Grigoris V. Karamasis, Yoshiaki Kawase, Nicholas M. Robinson, Andrew S.P. Sharp, Javier Escaned, Justin E. Davies

**Affiliations:** a Hammersmith Hospital, Imperial College NHS Trust, London, United Kingdom; b St. Marianna University School of Medicine, Kawasaki, Japan; c Essex Cardiothoracic Centre, Basildon, United Kingdom; d Anglia Ruskin School of Medicine, Chelmsford, Essex, United Kingdom; e Geneva University Hospital, Geneva, Switzerland; f Hôpital Sacré-Coeur de Montréal, Université de Montréal, Montréal, Canada; g Royal North Shore, Sydney, Australia; h Banner Heart Hospital, Mesa, Arizona; i Gifu Heart Centre, Gifu, Japan; j Tsukuba Medical Center Hospital, Tsukuba, Japan; k Fukuyama Cardiovascular Hospital, Fukuyama, Japan; l Wakayama Medical University, Wakayama, Japan; m Toda Central General Hospital, Toda, Japan; n St. Marianna University School of Medicine Yokohama City Seibu Hospital, Yokohama, Japan; o Hospital Clinico San Carlos, Madrid, Spain; p Kanazawa Cardiovascular Hospital, Kanazawa, Japan; q Okaya City Hospital, Okaya, Japan; r Royal Devon and Exeter Hospital, Exeter, United Kingdom

**Keywords:** artificial intelligence, coronary physiology, iFR, instantaneous wave-free ratio, percutaneous coronary intervention, AI, algorithmic interpretation, HT, heart team, iFR, instantaneous wave-free ratio, IQR, interquartile range, PCI, percutaneous coronary intervention

## Abstract

**Objectives:**

The aim of this study was to investigate whether algorithmic interpretation (AI) of instantaneous wave-free ratio (iFR) pressure-wire pull back data would be noninferior to expert human interpretation.

**Background:**

Interpretation of iFR pressure-wire pull back data can be complex and is subjective.

**Methods:**

Fifteen human experts interpreted 1,008 iFR pull back traces (691 unique, 317 duplicate). For each trace, experts determined the hemodynamic appropriateness for percutaneous coronary intervention (PCI) and, in such cases, the optimal physiological strategy for PCI. The heart team (HT) interpretation was determined by consensus of the individual expert opinions. The same 1,008 pull back traces were also interpreted algorithmically. The coprimary hypotheses of this study were that AI would be noninferior to the interpretation of the median expert human in determining: 1) the hemodynamic appropriateness for PCI; and 2) the physiological strategy for PCI.

**Results:**

Regarding the hemodynamic appropriateness for PCI, the median expert human demonstrated 89.3% agreement with the HT in comparison with 89.4% for AI (p < 0.01 for noninferiority). Across the 372 cases judged as hemodynamically appropriate for PCI according to the HT, the median expert human demonstrated 88.8% agreement with the HT in comparison with 89.7% for AI (p < 0.0001 for noninferiority). On reproducibility testing, the HT opinion itself changed 1 in 10 times for both the appropriateness for PCI and the physiological PCI strategy. In contrast, AI showed no change.

**Conclusions:**

AI of iFR pressure-wire pull back data was noninferior to expert human interpretation in determining both the hemodynamic appropriateness for PCI and the optimal physiological strategy for PCI.

Revascularization in stable coronary artery disease should be performed only for ischemia-producing coronary lesions 1, 2, 3, 4. Physiological measurements obtained using a coronary pressure-wire permit the identification of myocardial ischemia on a per vessel basis (5). Consequently, coronary physiology is recommended in international treatment guidelines 6, 7, 8 to guide revascularization decision making.

In addition to vessel-level ischemia detection, under resting conditions, a coronary pressure wire can also be used to produce an instantaneous wave-free ratio (iFR) pressure-wire pull back trace: a longitudinal assessment of coronary pressure loss along the length of a coronary artery. Such a trace permits the identification of lesion-level ischemia, as well as the ability to predict the physiological outcome following a proposed percutaneous coronary intervention (PCI) revascularization strategy (9). However, in the absence of clinical outcome data, a definitive interpretation of iFR coronary pressure-wire pull back data is lacking. Individual interpretation of coronary pressure-wire pull back data is complex, subjective, and dependent on the physiological expertise of the operator.

Algorithmic interpretation (AI) of coronary pressure-wire pull back data may help circumvent these limitations. Within this study, we aimed to determine if AI of iFR coronary pressure-wire pull back data could provide a standardized alternative to expert-level human interpretation. The coprimary hypotheses of this study were that AI would be noninferior to the interpretation of the median expert human in determining: 1) the hemodynamic appropriateness for PCI; and 2) the physiological PCI strategy, compared with the expert heart team (HT) opinion.

## Methods

### Study population

Six cardiac centers (St. Marianna University School of Medicine Yokohama City Seibu Hospital, Toda Central General Hospital, Okaya City Hospital, Kanazawa Cardiovascular Hospital, Hospital Clinico San Carlos, and Hammersmith Hospital) contributed retrospectively acquired iFR coronary pressure-wire pull back traces to a stable coronary artery disease all-comers clinical registry. No angiographic, clinical circumstances, or inclusion or exclusion criteria were used in the selection process. All cases were acquired by operators using manual pull back. Each pull back tracing was anonymized to prevent the identification of patient-specific data. Because no exclusion criteria were applied, traces were included regardless of whether there was evidence of pressure-wire drift or measurement artifact. The investigator core laboratory determined the technical quality of each pull back tracing. Measurement artifact was determined by the presence of artifact in either the iFR pull back curve or electrocardiographic trace. The expert graders were blinded to the core laboratory’s classification of technical quality. This study received ethical approval from the local ethics committee at each participating center.

### Expert human interpretation

**Figure 1:**
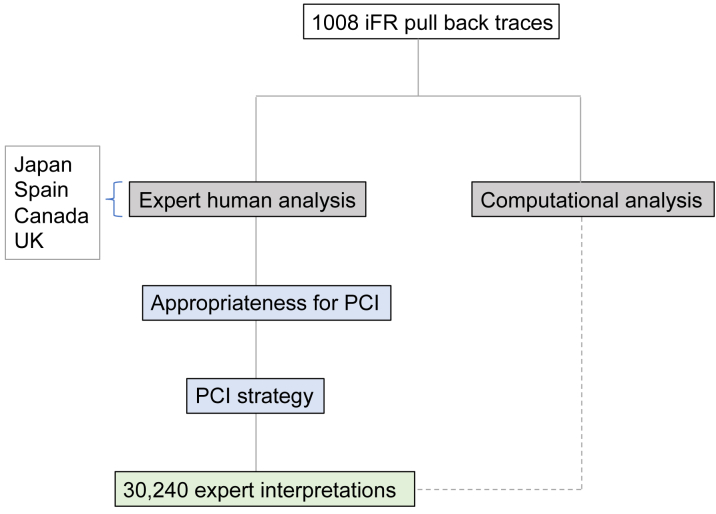
Study Design iFR = instantaneous wave-free ratio; PCI = percutaneous coronary intervention.

The study design is displayed in Figure 1. Fifteen interventional cardiologists with significant experience in the acquisition and interpretation of coronary physiology data evaluated 1,008 coronary pressure-wire pull back traces. Of these 1,008 coronary pressure-wire pull back traces, 691 were unique traces. The remaining 317 traces (31.4%) were duplicates, presented as new to each expert, to allow the assessment of intraobserver variability.

Using a dedicated online web portal system (Thalamus AI, Birmingham, United Kingdom), experts interpreted each trace independently, in a randomized order, without access to the coronary angiogram or additional clinical information. For each trace, experts were asked to determine the hemodynamic appropriateness for PCI. No specific criteria were provided to the experts to guide their decisions of hemodynamic appropriateness for PCI. In cases deemed hemodynamically appropriate for PCI, experts were further asked to determine their physiological strategy for PCI. This involved annotation on the pressure-wire pull back trace of the location(s) of physiologically significant lesion(s) the operator believed should be revascularized by PCI (Online Figure 1). This 2-step approach was used to replicate the cognitive steps involved in iFR-guided, ischemia-driven revascularization decision making. Specifically, the first step encapsulated the decision of whether revascularization should be performed on the basis of the iFR value, and in such cases, the second step encapsulated the decision of what the interventional strategy should be to obtain an optimal physiological outcome.

### Determining the HT interpretation

**Figure 2:**
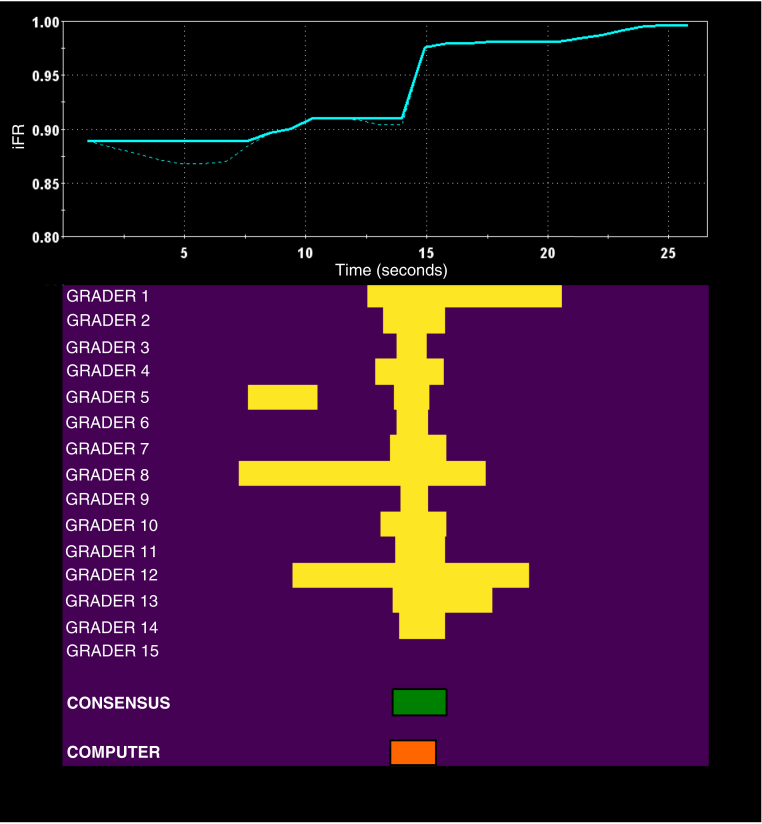
Determining the Consensus Opinion Shown is the coronary pressure-wire pull back trace **(blue line, top)**, the segment(s) of the pull back trace annotated for percutaneous coronary intervention (PCI) by the individual expert humans (**yellow blocks**, 1 row for each expert, **bottom**), the consensus expert human interpretation **(green block, bottom)**, and the algorithmic interpretation **(orange block, bottom)**. The PCI strategy consensus expert interpretation was created from segment(s) of the pull back trace that at least 50% of the individual expert humans had annotated for PCI. iFR = instantaneous wave-free ratio.

In the absence of clinical outcome data to provide a definitive interpretation of coronary pressure-wire pull back data, consensus expert opinion was used to determine the HT interpretation. Following grading, the annotated pressure-wire pull back traces were exported from the web portal and analyzed. For each of the 1,008 pull back cases, the HT PCI strategy was created by annotating only portions of the coronary pressure-wire pull back trace in areas in which the majority of the human experts (8 of 15) had indicated those areas for PCI (Figure 2).

### AI

The same 1,008 coronary pressure-wire pull back traces were then analyzed by a computer using computer algorithms to identify lesions on the pull back trace suitable for PCI. This AI considered information including the distal iFR value, the presence of physiologically significant pressure-wire drift, a Savitzky-Golay-filtered derivative (10) of the pull back trace, and the change in iFR value over any identified discrete lesions. Additional details regarding the development of the algorithm are provided in Online Appendix 1. The output from the algorithm was then compared with that of the HT interpretation and those of the individual experts. The algorithm was able to process 1.33 iFR pull-back traces per second.

### Study hypotheses

AI, individual expert human interpretation, and median expert human interpretation (i.e., the single human grader in the middle of the distribution of grader accuracies) were assessed against the HT interpretation. The coprimary hypotheses were: 1) noninferiority of algorithmic versus the median expert human interpretation for determining the hemodynamic appropriateness for PCI (the noninferiority margin was 5%); and 2) noninferiority of algorithmic versus the median expert human interpretation for determining the PCI strategy, compared with the expert HT opinion (the noninferiority margin was 5%).

### Statistical analysis

Categorical variables are expressed as numbers and percentages. Continuous variables are expressed as mean ± SD or median (interquartile range [IQR]), as appropriate. For the revascularization decision-making endpoint, the McNemar exact test was used with noninferiority analysis obtained through balanced reclassification of cases. For the PCI strategy endpoint, the accuracy (percentage of the pull back classified in agreement with the HT as PCI or non-PCI) for each case for both the computer algorithm and median expert human were compared. Additional details regarding the statistical analysis, noninferiority margin rationale, and sample size calculations are provided in Online Appendix 2. All analyses were performed using R version 3.2.1 (R Foundation for Statistical Computing, Vienna, Austria).

## Results

### Study population

The baseline demographic characteristics of the patients are shown in Table 1. The mean age of the patients was 65.4 years, and 74% were men. The physiological characteristics of the vessels are shown in Table 2. The median iFR value was 0.87 (IQR: 0.81 to 0.91). The distribution of iFR values is shown in Online Figure 2.

**Table 1:** Baseline Characteristics of the Patients (n = 640)

Age, yrs	65.4 ± 10.6
Male	473 (73.9)
Hypertension	442 (69.1)
Dyslipidemia	387 (60.5)
Diabetes mellitus	236 (36.9)
Chronic kidney disease	98 (15.3)
Current or ex-smoker	253 (39.5)
Family history of CAD	99 (15.5)
Previous myocardial infarction	148 (23.1)
Impaired LV function (EF <30%)	33 (5.2)

Values are mean ± SD or n (%).

CAD = coronary artery disease; EF = ejection fraction; LV = left ventricular.

**Table 2:** Physiological Characteristics of the Vessels (n = 691)

Distal iFR value	
Median	0.87
Interquartile range	0.81–0.91
Proximal iFR value	
Median	0.99
Interquartile range	0.98–1.01
Vessel evaluated	
Total	691 (100%)
Left anterior descending coronary artery	549 (79.5%)
Left circumflex coronary artery	72 (10.4%)
Right coronary artery	56 (8.1%)
Other	14 (2.0%)
Hemodynamic significance	
Distal iFR ≤0.89	470 (68.0%)
Distal iFR >0.89	221 (32.0%)
Significant pressure-wire drift	
Total	217 (31.4%)
Proximal iFR <0.98	147 (21.3%)
Proximal iFR >1.02	70 (10.1%)

iFR = instantaneous wave-free ratio.

Prior to correcting for pressure-wire drift, across the 691 unique pressure-wire pull back traces, 470 traces (68.0%) demonstrated distal iFRs ≤0.89, with 221 traces (32.0%) demonstrating distal iFRs >0.89. AI detected physiologically significant pressure-wire drift in 217 traces (31.4%), 70 (32.3%) with positive drift with proximal iFRs >1.02 and 147 (67.7%) with negative drift with proximal iFRs <0.98. After correcting for pressure-wire drift, 64 cases (9.3%) changed hemodynamic classification, with 45 cases (6.5%) becoming hemodynamically nonsignificant and 19 cases (2.7%) becoming hemodynamically significant.

### Hemodynamic appropriateness for PCI

**Figure 3:**
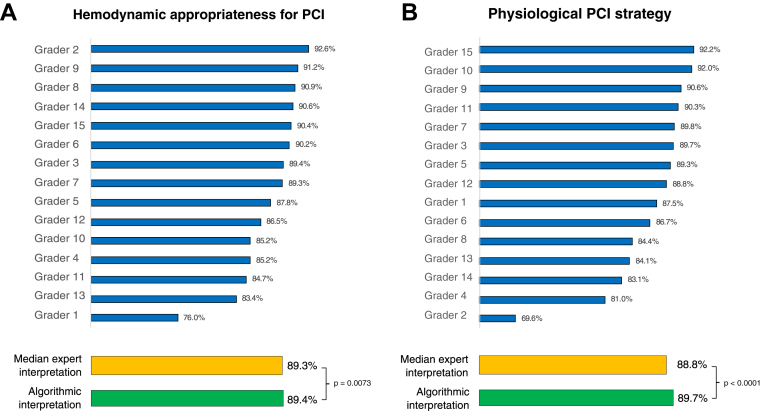
Percentage Agreement With Consensus Opinion Shown is the percentage agreement between individual expert humans **(blue bars)**, the median expert human **(orange bar)**, and algorithmic interpretation **(green bar)** compared with the consensus expert human interpretation for **(A)** the appropriateness for percutaneous coronary intervention (PCI) and **(B)** the PCI strategy.

Across all 691 unique cases, the median expert human agreed with the HT in 617 of 691 cases (89.3%; Cohen’s kappa = 0.78). AI agreed with the HT in 618 of 691 cases (89.4%; Cohen’s kappa = 0.79) and was noninferior to the median expert human (p = 0.0073; Cohen’s kappa = 0.70) (Figure 3A).

**Figure 4:**
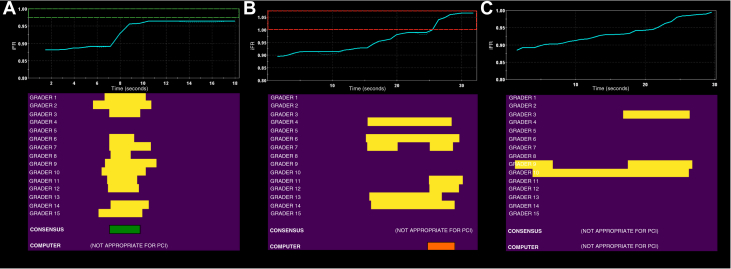
Appropriateness for Percutaneous Coronary Intervention **(A)** Disagreement between the consensus expert human interpretation (appropriate for percutaneous coronary intervention [PCI]) and algorithmic interpretation (inappropriate for PCI) because of the presence of physiologically significant negative pressure-wire drift. **(B)** Disagreement between the consensus expert human interpretation (inappropriate for PCI) and algorithmic interpretation (appropriate for PCI) because of the presence of physiologically significant positive pressure-wire drift. **(C)** Agreement between the consensus expert human interpretation (inappropriate for PCI) and algorithmic interpretation (inappropriate for PCI) because of the presence of a physiologically diffuse pattern of coronary artery disease, despite hemodynamic significance. iFR = instantaneous wave-free ratio.

Across the 372 cases that the HT determined were hemodynamically appropriate for PCI, 14 cases (3.8%) had hemodynamically nonsignificant physiology due to physiologically significant pressure-wire drift not corrected for by the consensus (Figure 4A). In contrast, using AI, there were no cases in which PCI was determined appropriate for hemodynamically nonsignificant physiology, as pressure-wire drift was always identified by the computer.

There were 319 cases that were determined as not hemodynamically appropriate for PCI according to the HT. Of these, 86 cases (27.0%) had hemodynamically significant physiology (Figure 4B) that was not identified by the HT. In contrast, there were 296 cases that were determined as not appropriate for PCI according to AI. Of these, 49 cases (16.6%) had hemodynamically significant but diffuse nonfocal physiology (Figure 4C).

### Physiological PCI strategy

The HT determined 372 of the 691 unique cases (53.8%) as hemodynamically appropriate for PCI. Across these cases, the median expert human demonstrated 88.8% agreement with the HT regarding the physiological PCI strategy. AI demonstrated 89.7% agreement (IQR: 85.6% to 97.2%) on the same cases and was noninferior to the median expert human (p < 0.0001) (Figure 3B).

### Intraobserver variability

Of the 1,008 traces reported by the expert humans, 317 traces were duplicates. Across the expert humans, the reproducibility of determining the hemodynamic appropriateness for PCI varied between 81.6% and 93.7%. The reproducibility of the median expert human was 90.8%, meaning that expert human decision making changed in ∼1 in 10 cases when reviewing the same coronary pressure-wire pull back data. In contrast, AI was identical on repeated testing of the appropriateness for PCI.

Of the 317 duplicate traces, 174 cases were determined to be hemodynamically appropriate for PCI according to the HT. Across the expert humans, the reproducibility of determining the physiological PCI strategy varied between 82.9% and 92.4%. The reproducibility of the median expert human was 90.4%. In contrast, AI was identical on repeated testing of PCI strategy.

### Performance in the borderline iFR zone

Borderline iFR values (0.88 to 0.92) were present in 228 of the 691 unique cases. For the hemodynamic appropriateness for PCI in the borderline iFR zone, the median expert human agreed with the HT in 193 of 228 cases (84.6%). The algorithm was numerically but statistically nonsignificantly superior to this, with accuracy of 85.5%. For PCI strategy in the borderline iFR zone, the median expert human demonstrated 89.3% agreement with the HT (IQR: 83.3% to 97.5%). AI demonstrated 91.4% agreement (IQR: 89.3% to 97.7%) on the same cases and was noninferior to the median expert human (p < 0.0001).

### Performance in physiologically tandem lesions

Analysis of the physiological PCI strategy endpoint in tandem lesions yielded 185 cases for analysis. The median human accuracy was 87.6% (IQR: 81.0% to 96.4%). The algorithm was numerically but statistically nonsignificantly superior to this, with accuracy of 87.8% (IQR: 83.1% to 94.0%).

### Disagreement between AI and HT interpretation

Across the 691 unique cases, there were 73 cases (10.6%) in which AI and HT interpretation disagreed on the hemodynamic appropriateness for PCI. Of these, 48 cases (65.8%) were when the HT interpretation was to defer, whereas the algorithm indicated hemodynamic appropriateness for PCI. In 15 of these cases (31.3%), this recommendation was explained by the presence of significant pressure-wire drift that was unrecognized by the HT. In the remaining 33 cases (68.7%) in which the HT interpretation was to defer but the algorithm chose to perform PCI, there was always at least 1 human who agreed with the algorithm.

Conversely, there were 25 cases (34.2%) in which the HT interpretation was to perform PCI, whereas the algorithm indicated deferral. In 13 of these cases (52.0%), this recommendation was explained by the presence of pressure-wire drift that was unrecognized by the HT. In the remaining 12 cases (48.0%) in which the HT interpretation was to perform PCI but the algorithm chose to defer, there was always at least 1 human who agreed with the algorithm. Details regarding disagreement between AI and HT interpretations within the borderline iFR zone (0.88 to 0.92) are provided in Online Appendix 3.

### Summary of implications

Tabulated summaries of the overall implications of algorithmic versus HT interpretation of iFR pressure-wire pull back data are presented in Online Tables 1 and 2. In summary, across all 691 iFR pull back traces, compared with the HT consensus, AI recommended a greater rate of revascularization (53.8% vs. 57.2%; p < 0.01) and a greater number of stented segments per pull back trace (1.28 vs. 1.48; p < 0.0001).

## Discussion

In this study we demonstrated that AI of iFR pressure-wire pull back data was noninferior to the interpretation of the median expert human in determining both the hemodynamic appropriateness for PCI and the physiological PCI strategy when judged against an expert HT opinion. AI correctly interpreted physiologically significant coronary pressure gradients and modified treatment accordingly in the presence of pressure-wire drift (Central Illustration).

**Central Illustration:**
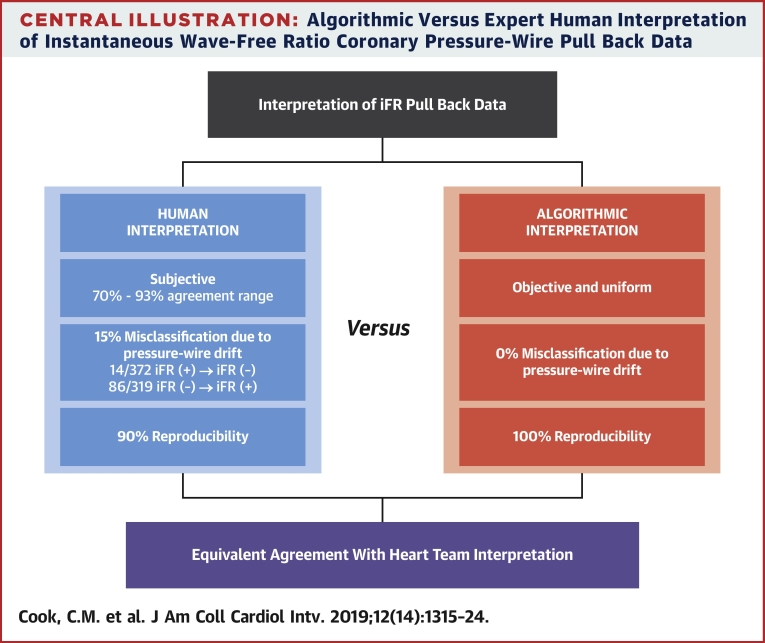
Algorithmic Versus Expert Human Interpretation of Instantaneous Wave-Free Ratio Coronary Pressure-Wire Pull Back Data

### HT decision making

Group decision making has become commonplace in cardiology, with the role of the HT well established in the management of complex clinical decision making 11, 12, 13. Additionally, group decision making can be valuable in areas of medicine in which the optimal treatment approach remains uncertain because of a lack of clinical outcome data. In that regard, the interpretation of coronary pressure-wire pull back data is often complex, and a treatment plan must usually be decided upon instantaneously. However, practically speaking, HT opinion for this task is rarely available. As such, having a computer-aided tool instantaneously capable of providing high agreement with expert HT opinion could provide a useful adjunct to clinical decision making, lessening the burden of ad hoc decision making placed upon individual physicians.

### Disagreement between AI and HT interpretation

In the absence of clinical outcome data, there currently exists no definitive gold standard for interpreting iFR pressure-wire pull back data. Accordingly, in this study, we have assessed AI by judging it against the expert HT opinion. However, this approach may actually bias the results in favor of an expert human over the algorithmic approach, as each individual human’s decision will have contributed toward determining the HT opinion for that case. Furthermore, in a number of cases in which the AI and the HT interpretation differed (thereby reducing the accuracy of the AI), the HT consensus interpretation itself could be considered to be flawed. This occurred primarily because of the inability of the HT to recognize hemodynamically significant pressure-wire drift.

### Uniform implementation of treatment guidelines

Advantages of an algorithmic approach are that it provides uniform implementation of treatment guidelines independent of environmental and sensory limitations that may present pitfalls for coronary physiology-guided revascularization decision making. Common examples of this include the failure of the human operator to notice and correct for physiologically significant pressure-wire drift or interpret data in the presence of measurement artifact. The failure to acknowledge pressure-wire drift in particular can lead to large changes in the hemodynamic significance of stenoses in borderline zone iFR values (14). In this study, we have shown that an algorithmic approach can ensure that no hemodynamically nonsignificant lesions receive PCI, while also reducing the number of hemodynamically significant lesions that are deferred from PCI.

Additionally, there was variation in how individual expert humans performed in determining the hemodynamic appropriateness of PCI compared with determining the physiological PCI strategy. The human expert with the highest agreement with consensus for the hemodynamic appropriateness of PCI subsequently demonstrated the lowest agreement with consensus for the PCI strategy (Figure 3). This indicates that at the 2 levels of expertise required for the interpretation of coronary pressure-wire pull back data, these levels of expertise can be different.

Last, an algorithmic approach allows modifications to be made in light of new evidence and updated guideline recommendations. Using such a system could help improve the overall appropriateness of revascularization decision making (7) and ensure that the latest guidelines and evidence propagates immediately to all physicians and patients.

### Study limitations

In this study, experts were asked to determine their optimal physiological PCI strategy solely on the basis of the interpretation of coronary pressure-wire pull back data. Accordingly, we did not capture additional data that may have influenced these decision (e.g., the physiological length of disease or diffuseness of disease). Furthermore, it is likely that such decisions may be modified when pull back data are subsequently integrated with the coronary angiogram and individual patient characteristics. Because these factors were not available, we are unable to determine how they may have modulated the interpretation of physiological data with regard to revascularization decision making.

The majority of pressure-wire pull back traces in our all-comers clinical dataset were from left anterior descending vessels. This high prevalence of left anterior descending vessels (79.5%) is similar to that observed in other nonselected real-world physiological datasets (15). Furthermore, neither the human graders nor the algorithm was ever aware which vessel the iFR measurements were performed in.

Although in this study we applied our AI to iFR pull back data alone, should the post-PCI predictive accuracy of the resting indexes such as the diastolic pressure ratio be demonstrated, our algorithmic approach could be applied. This would help ensure broader application of computer-assisted interpretation of physiological data, independent of the provider of coronary catheter laboratory equipment.

Not captured within our study methodology are the additional barriers that exist to iFR pull back data interpretation in real-world physical environments. Such barriers include, for example, limitations of the human visual system to discern small hemodynamic gradients on a physiology screen that may be positioned at a distance away from the operator. Additionally, the very nature of performing an invasive physiological assessment necessitates multitasking of the operator, whose ability to focus solely on interpretation of hemodynamic data is limited. However, within our study, these types of barriers were not replicated, and thus their influence on human decision making remains unmeasured. Although speculative, it is possible that true real-world interpretation of coronary pressure-wire data may be more variable and heterogenous than that recorded within our study.

Last, because of the retrospective nature of our dataset, the actual clinical decisions made at the time of iFR pull back measurement were neither recorded nor informed by knowledge of the AI.

## Conclusions

AI of iFR pressure-wire pull back data provided a standardized interpretation that was automatically corrected for the presence of pressure-wire drift. When judged against an expert HT opinion, AI was noninferior to that of the median expert human in determining both the hemodynamic appropriateness for PCI and the PCI strategy.

Perspectives **WHAT IS KNOWN?** In the absence of clinical outcomes data, a definitive interpretation of iFR pull back data is lacking. Individual interpretation of coronary pressure-wire pull back data can be complex, subjective, and dependent on the physiological expertise of the operator.

**WHAT IS NEW?** AI data provided a standardized and instantaneous interpretation of iFR pressure-wire pull back that was automatically corrected for the presence of pressure-wire drift. When judged against an expert HT opinion, AI was noninferior to that of an expert human.

**WHAT IS NEXT?** This study investigated the AI of only 1 aspect of coronary revascularization decision making (i.e., iFR pressure-wire pull back data). Future innovations should be tailored toward developing systems that can analyze and interpret the multiple additional factors that influence human clinical decision making (e.g., angiographic characteristics, patient-specific factors).
