# Supplementary material for: Algorithmic Versus Expert Human Interpretation of Instantaneous Wave-Free Ratio Coronary Pressure-Wire Pull Back Data
Source: JACC Cardiovasc Interv. 2019 Jul 22;12(14):1315–24. doi: 10.1016/j.jcin.2019.05.025 (PMC6645043; doi:10.1016/j.jcin.2019.05.025)
Supplement: Online Data [file mmc1.docx]

# **Appendix 1**

This algorithmic interpretation was developed using a separate pilot dataset of 160 coronary pressure-wire pullback traces which were annotated by the study team, with parameters optimized to maximise agreement between the algorithm and human annotations. Optimization involved identification of clinical cut points for the treatment or deferral of coronary artery disease (iFR ≤0.89), after accounting for pressure-wire drift within the pressure wire pullback trace. The computer was trained to recognise focal from diffuse areas by iterative training (Equation 1), and development of a series of co-efficients which represent a mathematical expression of the physiological disease pattern. These governed the weighing of several parameters including the gradient of the pullback trace in iFR units per second, the total iFR drop over a discrete lesion, the length of stent landing zones, and the distance between any serial lesions. The study team involved in generating this training data did not contribute towards the dataset of 1008 cases used for algorithm validation. Because this study involves the analysis of physiology traces alone, without accompanying coronary angiograms, the definition of a tandem lesion was determined physiologically; where the algorithm identified at least two discrete pressure gradients.

The derivative used for deciding the focality and significance of lesions is the change in pressure ratio over time (iFR units per second). The derivative was filtered over time due to the use of a Savitzky-Golay filter. A window length of 21 datapoints for this calculation was used.

.

**Appendix 2**

Data were normalized using Tukey's Ladder of Powers and a paired t-test was performed to test for a difference in accuracy between the algorithm and median human across cases. For sample size calculations we assumed 75% accuracy for both the median human and algorithm for the PCI strategy endpoint with a standard deviation of 15%, meaning 250 pullbacks would need to be analyzed by both to provide 95% power to detect 5% non-inferiority at the 5% significance level. Assuming only 50% of cases are suitable for PCI, this means 500 unique pullbacks would need to be analyzed. Then, assuming 85% accuracy for both the median human and algorithm for the revascularization decision-making endpoint (corresponding to a p_00_, p_10_, p_01_ and p_11_ of 0.76, 0.09, 0.09 and 0.06 respectively), the 500 patient sample size above would provide 95% power to detect 5% non-inferiority (performed by balanced reclassification) at the 5% significance level. Like all non-inferiority margins, this was chosen as a balance of judgement, especially given no prior information was available explaining the correlation between algorithms and humans. In this study, we predicted an 85% accuracy for both the algorithm and humans at the strategy endpoint. This corresponded to a 18% disagreement rate (76% correct agreement; 6% incorrect agreement). A 5% difference favoring the human would correspond to an 11.5% algorithm error rate versus 6.5% human error rate. The study team felt this represented the upper barrier of what was acceptable, as any higher would correspond to the human error rate being half of that of the algorithm. Applicable tests were 2 tailed and p < 0.05 was considered statistically significant.

**Appendix 3**

Within the borderline iFR zone (0.88-0.92), there were 33 cases of disagreement. In 24 (72.7%) of these cases, the HT interpretation was to defer, whilst the algorithm indicated PCI. In 11 (45.8%), this recommendation was explained by the presence of pressure-wire drift that was unrecognized by the Heart Team. In the remaining 13 cases, there was at least one human expert there was always at least one human who agreed with the algorithm. In the 9 (27.2%) cases where the algorithm deferred but the HT interpretation was for PCI, 4 (44.4%) of these are again explained by the presence of pressure-wire drift that was unrecognized by the Heart Team.

**Appendix 4**

**Table 1: Summary of implications I – Decisions to revascularize and number of stented segments**

| **Revascularization decisions (n = 691)** | | | |
| --- | --- | --- | --- |
|  | Heart Team consensus | Algorithm | P value |
| Decision to revascularize | 372 (53.8%) | 395 (57.2%) | 0.0095 |
| No of stented segments (in those for revascularized) | 475 (1.28 stents per case) | 585 (1.48 stents per case) | p < 0.0001 |

**Table 2: Summary of implications II – Overall performance of algorithmic interpretation versus median expert human**

| **Decision endpoint by Heart Team decision (n = 691)** | | |
| --- | --- | --- |
| Heart Team revascularize (n=372) | Median human | Algorithm |
|  | PCI decision endpoint | |
|  | 92.7% | 93.3% |
|  | PCI strategy endpoint | |
|  | 88.8% | 89.7% |
| Heart Team defer (n=319) | Median human | Algorithm |
|  | 87.8% | 85.0% |

**Online Figure 1.**


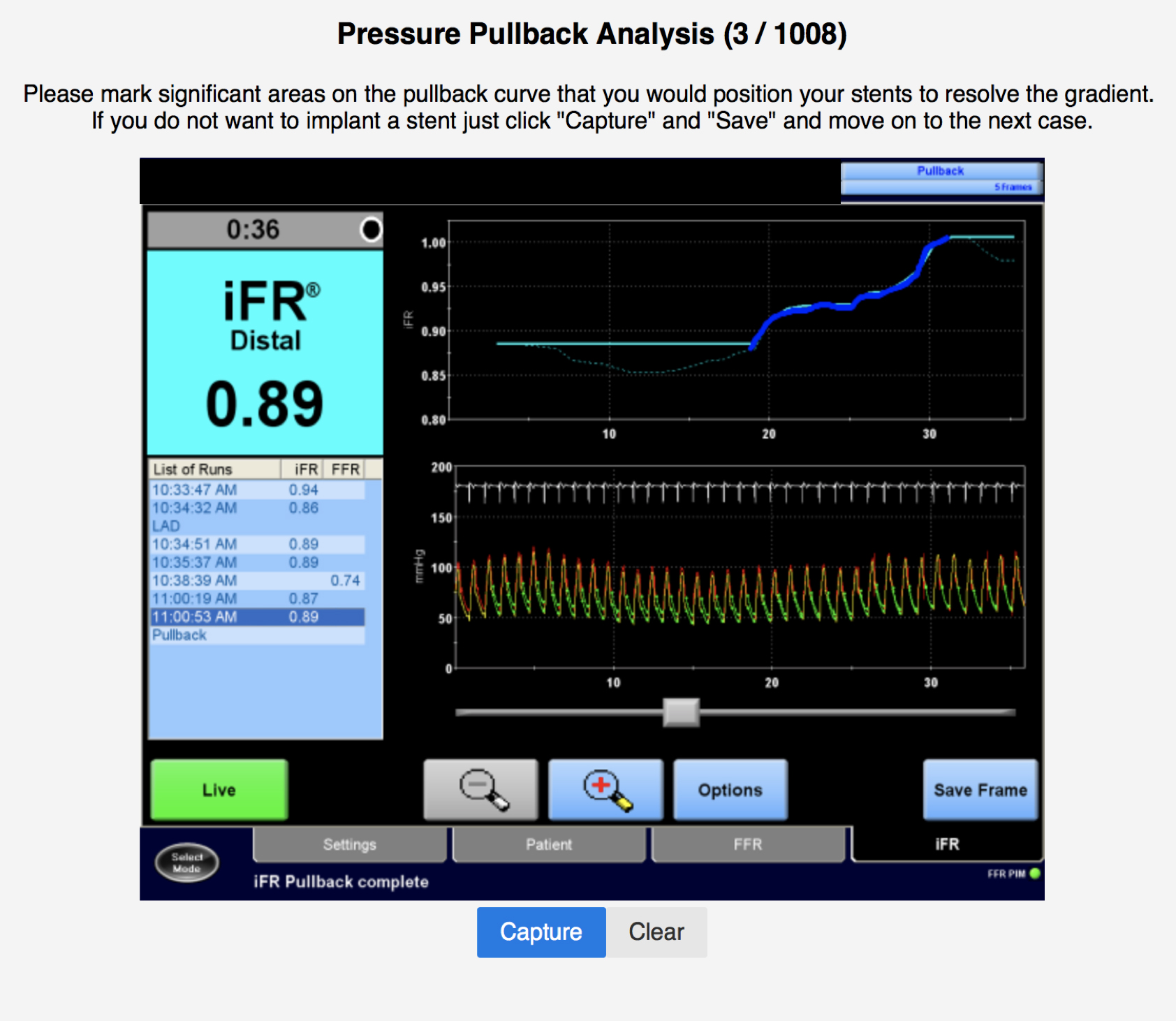


Screenshot from the web portal used by the experts to determine their PCI strategy. The expert has annotated on the pressure-wire pullback trace (dark blue) the location they believe should be revascularized by PCI.

**Online Figure 2.**

The vertical red line indicates the median iFR value. The vertical black line indicates the mean iFR value.
